# The application of nano-enrichment in CTC detection and the clinical significance of CTCs in non-small cell lung cancer (NSCLC) treatment

**DOI:** 10.1371/journal.pone.0219129

**Published:** 2019-07-25

**Authors:** Tengteng Wei, Donglin Zhu, Yong Yang, Guangda Yuan, Hongya Xie, Rongming Shen

**Affiliations:** Department of Thoracic Cardiovascular Surgery, Affiliated Suzhou Municipal Hospital of Nanjing Medical University, Suzhou, Jiangsu, China; Universitat de Barcelona, SPAIN

## Abstract

Circulating tumor cells (CTCs) are an independent prognostic marker in non-small cell lung cancer (NSCLC). CTC numbers are closely related to early diagnosis, clinical stage, therapy surveillance, and prognosis of NSCLC. We used a more efficient nano-enrichment method to detect CTCs in NSCLC patients and explored the clinical value of CTCs. The results showed that CTC numbers in stage IV cases were significantly higher than those in stage I, II or III cases. The number of CTCs in poorly-differentiated cases was significantly higher than that in well-differentiated cases. During six chemotherapy cycles, the average CTC number decreased from 5.8/7.5 ml in cycle #1 to 2.4/7.5 ml in cycle #4 and remained at almost the same level from 4 to 6 cycles. CTC numbers in patients with EGFR mutations was significantly higher than those in patients with no mutations. The average progression free survival (PFS) in the favorable group (CTC ≤ 5/7.5 ml) was 11.3 months, which was longer than that in the unfavorable group (CTC > 5/7.5 ml, 7.2 months). In conclusion, the assessment of NSCLC cannot be performed using a single CTC analysis. The clinical value is more significant in the continuous analysis of CTC data, as well as the cross-validation of other indexes and imaging results.

## Introduction

Lung cancer is currently the most common malignancy tumor with the highest morbidity and mortality. More than one million patients die from lung cancer annually in the world [1]. Approximately 80% of lung cancer patients are diagnosed with non-small cell lung cancer (NSCLC), with only 15% surviving for 5 years [2]. Surgery is not possible for most NSCLC patients as primary treatment, because these patients are often diagnosed at an advanced stage. Although the rates of detection and surgery success have improved with advancements in medical technology, the poor effects of adjuvant therapy and postoperative recurrence still have a substantial negative effect on overall treatment [3, 4]. If NSCLC could be diagnosed at an earlier stage, postoperative recurrence and effects of adjuvant therapy could be evaluated more comprehensively, and the overall treatment effect for NSCLC would be greatly improved, such as reduced mortality and improved quality of life.

Circulating tumor cells (CTCs) were first proposed by Ashworth in 1969 [5]. CTCs are tumor cells that enter the blood stream and are derived from tumor tissues. Most of the CTCs are eliminated by the human immune system, but a few of them with high activity and metastatic potential may survive in the circulatory system and clump together to form tiny cancerous nodules, which lead to metastasis and recurrence of cancer [5]. Studies have proven the existence of CTCs in the blood from patients with breast cancer, colorectal cancer, prostate cancer, gastric cancer and many other cancers, and CTCs are closely related to early diagnosis, clinical stage, therapy surveillance, and prognosis of cancer [6].

CTCs have been shown to be an independent prognostic marker in SCLC and NSCLC [7, 8]. At the same time, CTCs can also provide relevant information regarding tumor biological activity, and prognosis of patients with distant metastasis [9]. It has been reported that after chemotherapy for advanced lung cancer, CTC numbers can reflect subtle metastases and recurrent tumors which are difficult to detect by imaging [10]. Studies on the treatment of epithelial cancer and breast cancer have shown the effect of chemotherapeutic drugs on CTC apoptosis in vitro [11, 12]. In addition, CTCs are being gradually employed in the detection of mutations and rearrangements in common targeted genes (EGFR, ALK, KRAS, etc.) in tumor patients [13], and can indicate the effect of targeted drugs during the treatment of patients with EGFR mutations and incidence of drug resistance [14]. However, due to the limited detection of CTCs in NSCLC at present, there are relatively few studies and applications of CTCs, which cannot facilitate treatment and disease monitoring in NSCLC.

The extensive applications of CTC detection play a vital role in tumor study. The gold standard of CTC detection is the CellSearch System, which is based on the principle of specific immunological recognition of epithelial cell adhesion molecule (EpCam)-positive cells. The positive rate of CTCs is higher in other types of cancer than in NSCLC, which limits the use of CellSearch System in NSCLC [8, 15]. In addition, NSCLC lacks tumor marker protein in clinical detection [16]. Therefore, for more accurate and informative studies on NSCLC, a novel and more effective detection system for CTCs is crucial.

This study utilized a more efficient method based on nano enrichment to detect the CTCs in NSCLC patients [17]. Using data collected from 73 NSCLC patients, we explored the clinical value of CTCs in recurrence, metastasis, therapy surveillance and prognosis to provide better support for NSCLC therapy.

## Methods

### Patients and blood preparation

A total of 73 patients were enrolled in this study (mean age 63.7 years, range from 41 to 81 years). Written consent was obtained from all patients, and all protocols regarding the use of patient samples in this study were approved by the Ethics Committee of Suzhou Municipal Hospital. Chemotherapy was administered for 3 weeks per cycle. Depending on their condition, the patients received 4 to 6 rounds of chemotherapy. Blood (9 ml) was drawn from patients into CTC TransFix/EDTA vacuum blood collection tubes (CYTOMARK, UK) before each chemotherapy cycle (S1 Fig). Blood samples were stored at 4°C and processed within 48 h.

### Substrate preparation

A Corning 60-mm petri dish was washed for 12 min in a plasma cleaner. Then, 1 ml ethanol containing 3% APTES was added to the dish and cultured for 1 h in the dark. After that, APTES was absorbed, and the dish was washed 5–6 times with pure water. The dish was placed in an oven at 80°C for 1 h.

### CTC capture and detection

CTC capture and detection were performed according to the described workflow (S1 Fig). Approximately 7.5 ml blood samples was gently mixed according to the ratio of 1 ml blood to 10 ml 1x red blood cell lysis buffer and incubated at room temperature for 15 min. The mixture was centrifuged at 200 g for 5 min and the supernatant was discarded. The pellet was resuspended in 2 ml 1% FPBS and washed one time. Cells were added to the substrate and cultured at 37°C for 1 h. After that, the substrate with cells was transferred to 4°C for 10 min. The liquid was replaced by 4% paraformaldehyde (PFA) and incubated at 4°C for 10 min. Then, the 4% PFA was replaced by pre-cooled methanol and incubated at -20°C for 10 min. Then, methanol was discarded, and the substrate was washed three times with PBS.

Subsequently, 2% skim milk in PBS was added to the substrate for blocking. Then, 2% skim milk containing 1 μl anti-pan-cytokeratin-Cy5 and 1μl anti-CD45-FITC antibodies were added. After culturing at 4°C overnight, PBS was used to wash the substrates three times. DAPI was used to label the nuclei. The images of CTCs were captured by a Cytell cell imaging system. The treated samples were scanned by the high-throughput capture Cytell Cell Imaging System (GE Healthcare Life Sciences). Five hundred random scan points for each sample were captured using three different emission spectra [17]. DAPI+, CK+ and CD45- cells were identified as CTCs. The number of CTCs was counted by the supporting software.

### Cell culture and spiking test

H1975 cells (human non-small cell lung cancer cell line) were purchased from the Shanghai Institute of Cell Biology, Chinese Academy of Sciences, and cultured in a cell culture with incubator RPMI-1640 supplemented with 10% fetal bovine serum at 37°C with 5% CO_2_. For the spiking test, H1975 cells were separated by trypsinization and counted using a hemocytometer with trypan blue staining. To detect the proportion of tumor cells in urine, we spiked 50, 100, and 200 H1975 cells into 7.5 ml blood samples from healthy people. The results are shown in S2 Fig.

### EGFR and ALK mutation detection

The L858R mutation and the exon 19 deletion mutation of EGFR were detected by ARMS-PCR using multiple primers (S1 Table). PCR was performed in a 25 μl reaction tube containing 12.5 μl GoldStar TaqMan Mix (CWBiotech, China), 2 μl template DNA, 0.5 μl probe, 0.5 μl mixed primer, and 9 μl DI water. Amplification was carried out under the following conditions: 95°C for 5 min, 10 cycles at 95°C for 30 s, 62°C for 30 s without fluorescence acquisition, 35 cycles of 95°C for 30 s, and 58°C for 30 s with fluorescence acquisition (Ex 480 nm/Em 520 nm) at the end of each cycle. To determine the sensitivity of three PCR assays, wild-type EGFR DNA samples (wt-DNA) and DNA samples containing EGFR E19del or L858R mutation (mutant DNA) were prepared and first confirmed by Sanger sequencing. To test the concentration effect and MAF, we serially diluted the mutant DNA with deionized water or wt-DNA to create a sensitivity panel consisting of 0.01%, 0.1%, 1%, and 10% mutant DNA. Two negative controls (either without DNA template or with wt-DNA) and mutation positive controls were also included.

ALK rearrangements were detected by fluorescent in situ hybridization (ALK Mutation Detection Kit, Linked-Biotech Pathology, China) according to the instruction manual. Samples were considered FISH-positive if more than 15% of the scored tumor cells had split one or both ALK 5' and 3' probe signals or had isolated 3' signals. Slides were evaluated independently by two experts who were blinded to the patient’s history and histologic findings.

### Statistics

GraphPad v6.0 was used for analyses. The results are presented as the mean ± SD. Differences between groups were calculated by Student’s t-tests, and p < 0.05 was considered statistically significant.

## Results

From February 2017 to July 2018, 73 patients with NSCLC were enrolled in this study. The patient characteristics are summarized in Table 1. Among them, 65 patients had chemotherapy indications and received chemotherapy. Thirty-seven patients experienced disease progression.

**Table 1:** Patient characteristics.

Characteristic	Total (N = 73)
Age—yr	
Mean	63.7
Range	41–81
Sex—no.(%)	
Male	41 (56.2)
Female	32 (43.8)
Stage—no.(%)	
Tis	9 (12.3)
I	24 (32.9)
II	23 (31.5)
III	12 (16.4)
IV	5 (6.8)
EGFR mutation—no.(%)	Total (N = 14)
Exon 19 deletion	5 (35.7)
L858R	2 (14.3)
No mutation	7 (50.0)

**Fig 1:**
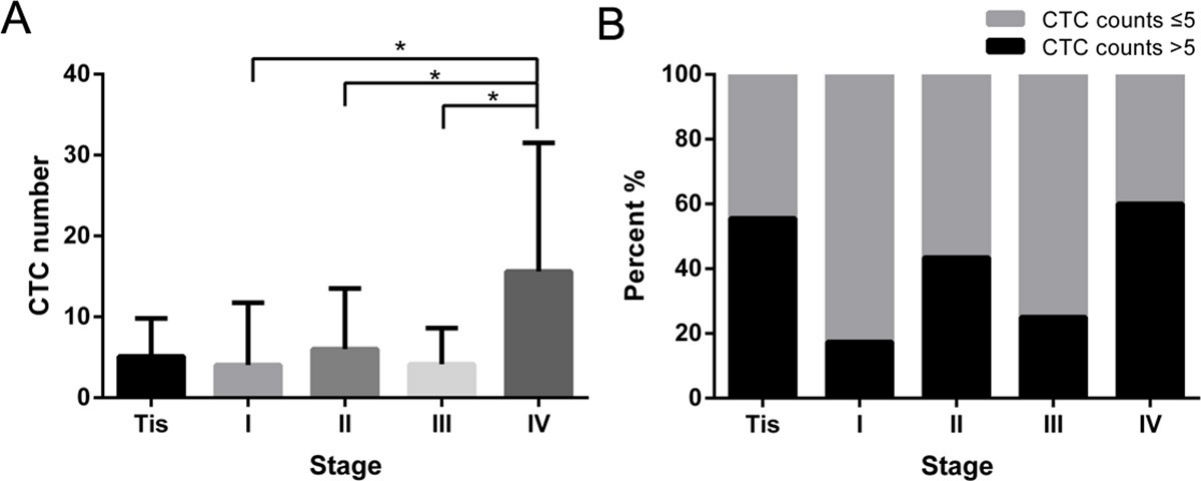
CTC numbers in each NSCLC stage. A. CTC numbers based on different NSCLC stages. B. Percentage of patients with CTC numbers greater than 5/7.5 ml or not. * p < 0.05.

The CTC numbers of NSCLC patients varied with different stages. The CTC number in stage IV disease was significantly higher than that in stage I, II or III disease (Fig 1A). Among the patients with CTC numbers greater than 5/7.5 ml, stage I accounted for the lowest proportion (17.4%), and stage IV accounted for the highest proportion (60%) of patients (Fig 1B).

**Fig 2:**
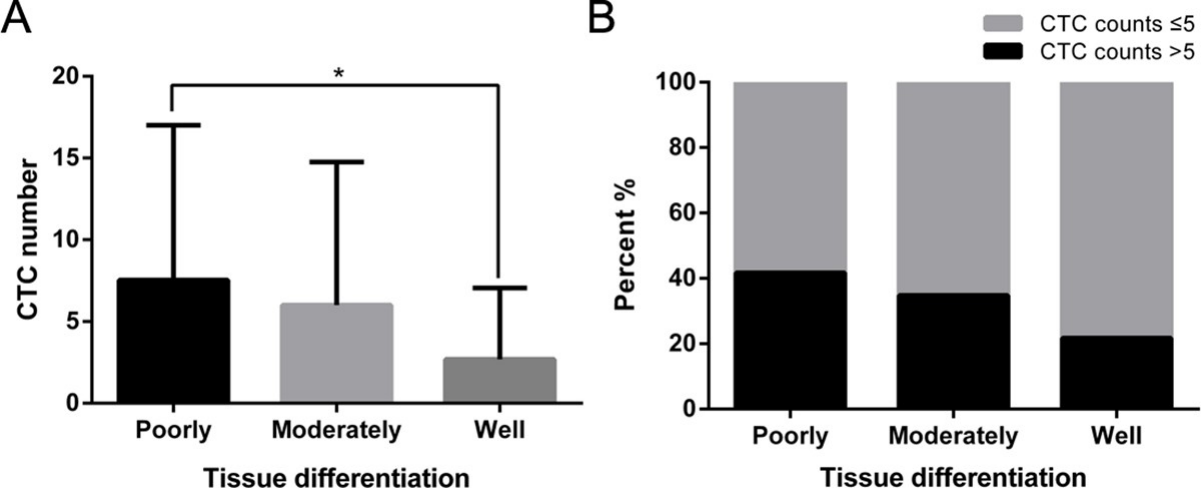
CTC numbers in patients based on tumor differentiation. A. CTC numbers in NSCLC patients based on different tumor differentiation. B. Percentage of patients with CTC more than 5/7.5 ml or not. * p < 0.05.

In colorectal cancer, Yang et al [18] reported that the number of CTCs in patients with poorly-differentiated tumors was significantly higher than that in patients with well-differentiated tumors. We obtained similar results in NSCLC (Fig 2A). Among them, patients with CTC greater than 5/7.5 ml accounted for 41.7% of poorly- differentiated and 21.7% of well-differentiated cases (Fig 2B).

**Fig 3:**
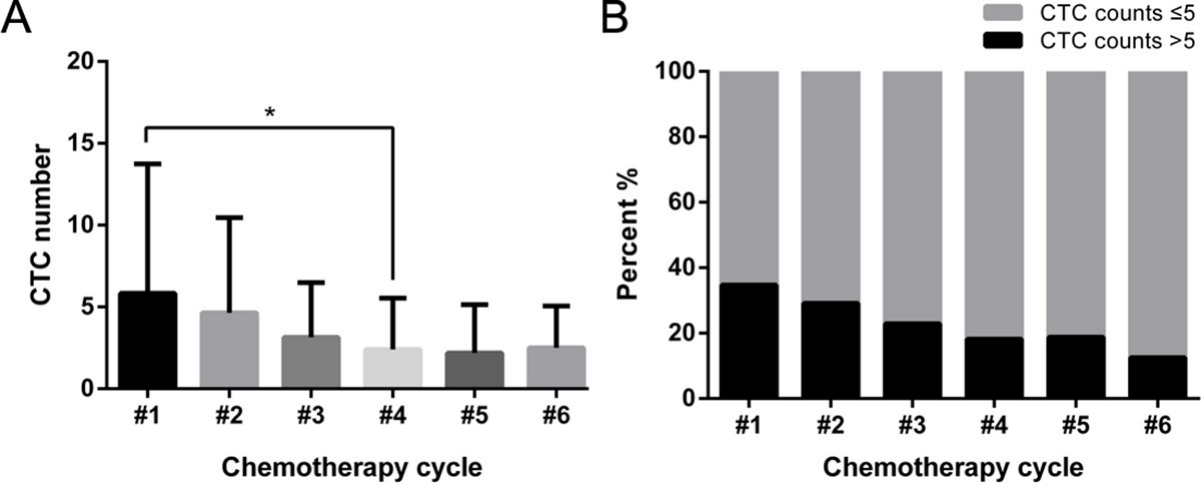
CTC numbers in patients undergoing chemotherapy cycles. A. CTC numbers of NSCLC patients based on different number of chemotherapy cycles. B. Percentage of patients with CTC numbers greater than 5/7.5 ml or not. * p < 0.05.

Chemotherapy is one of the major adjuvant treatments for patients with lung cancer after surgery. Chemotherapy is usually administered over several cycles and we collected blood samples from patients before each cycle was performed. We found that during the chemotherapy cycles, the average CTC numbers decreased from 5.8/7.5 ml in cycle #1 to 2.4/7.5 ml in cycle #4 (Fig 3A). The proportion of patients with CTC numbers greater than 5/7.5 ml also decreased (Fig 3B). These findings were consistent with routine imaging studies and patients’ self-perceptions.

**Fig 4:**
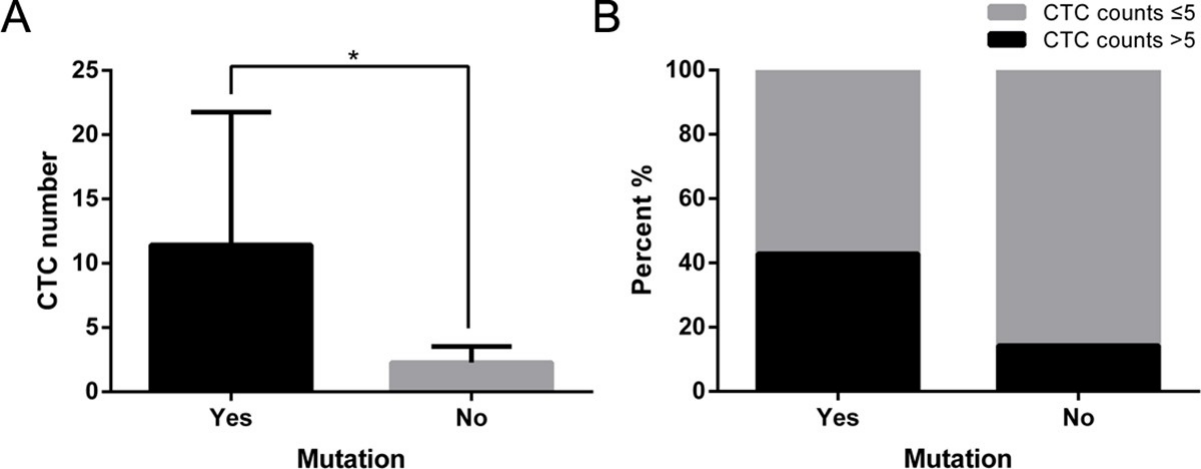
CTC numbers in patients with or without EGFR mutations. A. CTC numbers in NSCLC patients with or without EGFR mutations. B. Percentage of patients with CTC numbers greater than 5/7.5 ml or not. * p < 0.05.

With the widespread application of tyrosine kinase inhibitors (TKIs), especially EGFR-TKIs, the mutational status of EGFR has become increasingly important in recent years. We detected the EGFR mutational status in 14 patients, 7 of whom had an EGFR mutation (5 patients with E19del and 2 with L858R). Then we compared CTC numbers of patients with different EGFR mutational statuses. CTC numbers in patients with EGFR mutations were significantly higher than that in patients with no mutations (Fig 4A). We also found that the proportion of cases with CTC numbers greater than 5/7.5 ml was higher in patients with EGFR mutations than in patients without EGFR mutations (Fig 4B).

**Fig 5:**
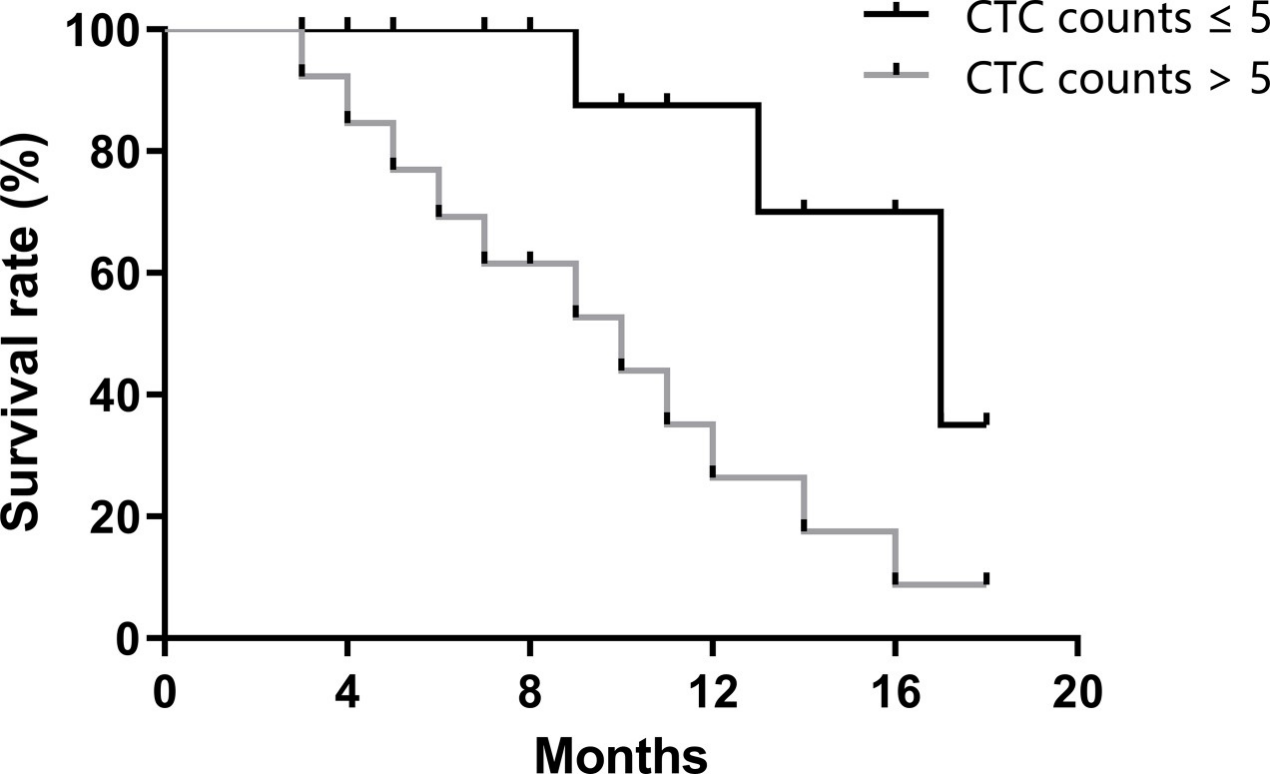
Progression-free survival analysis.

A total of 73 patients were divided into a favorable group (CTC ≤ 5/7.5 ml) and an unfavorable group (CTC numbers > 5/7.5 ml) [19, 20]. Then, we calculated the progression-free survival (PFS) of these patients. The average PFS of the favorable group was 11.3 months, while the average PFS of the unfavorable group was 7.2 months (Fig 5). Patients with CTC numbers lower than 5/7.5 ml had longer PFS. (p < 0.05)

## Discussion

The method used in this study has shown superiority in CTC detection [17]. This approach displayed a higher positive rate and accuracy than physical and biological isolation methods, as it was not limited to EpCam, which causes false negatives. It could also compensate for the limitation of physical properties, such as CTC deformability and friction. Among all 79 subjects, 43 were identified to have CTC for the first time in the CTC analysis and 24 had CTC numbers greater than 5/7.5 ml. We summarized the reported CTC analysis results in NSCLC patients in S3 Fig. CellSearch was the most popular method for CTC detection in the past few years. In addition, ISET and some non-commercial methods have been employed in CTC detection for NSCLC patients. The positive rates ranged from 13% [21] to 100% [22]. Our results (54.4%) represented the average CTC positive rate (54.2%). The average CTC numbers of the total cohort and CTC-positive cohort were 5.5/7.5 ml and 9.2/7.5 ml in our study, which were both near the middle of reported results.

Recurrence and metastasis of lung cancer occur via CTCs and seriously affect the survival of patients even after surgery. The key to improving prognosis is to use better approaches for surveillance. For patients with different stages of disease, the CTC number differs. Continuous detection of CTCs can offer valuable insights. Our data a significant increase in CTC number in stage IV cases. However, the CTC numbers of patients with stage Tis, I, II and III disease were not significantly different from one another. This result might be related to the low number of CTCs in early-stage cancer [8]. Furthermore, there is no linear correlation with clinical stage.

Some published papers have reported a linear correlation between CTC number and clinical stage [23]. Nevertheless, other published papers disagreed [15]. There is still a dispute in this regard. We will continue collecting samples and conducting further research.

The differentiation state of NSCLC influences recurrence and metastasis to some degree [24]. Within the same stage, poorly-differentiated NSCLC cases are more prone to recurrence and metastasis than well-differentiated cases [25]. Our study showed that poorly-differentiated NSCLC cases had more CTCs, while well-differentiated NSCLC cases had fewer CTCs. This result was consistent with the view above. Thus, patients with more CTCs should be more carefully monitored and recommended for further examination, such as imaging, or additional treatment.

To evaluate the role of CTCs in guiding NSCLC therapy, we continuously monitored CTC counts in patients before and after chemotherapy. The results showed that the CTC numbers decreased gradually during the four courses of chemotherapy, which was consistent with the imaging results. There was no difference in CTC numbers between the four-course and six-course treatment, suggesting that either the CTC number had decreased to the lowest level and the small discrepancy was unmeasurable; or the emergence of resistance reduced the effect of chemotherapy. Therefore, continuous monitoring and analysis of changes in CTC numbers is meaningful, and it can better discern the treatment effect and occurrence of resistance.

There have been an increasing number of studies on molecular targeting technologies over the past few years. Molecular targeted drugs, especially EGFR-TKIs, display the characteristics of good therapeutic effect and low rates of side effects, and have become the first-line drugs for treating NSCLC. The E19del and L858R mutation of EGFR can result in the sensitivity of tumor cells to EGFR-TKIs. A report proposed that CellSearch cannot provide background information on CTCs, but it can be used to isolate CTCs for targeted therapy, surveillance and drug resistance [26]. In some patients who cannot tolerate invasive examination or in a situation where obtaining tissue specimens is challenging, it is difficult to examine EGFR mutations and ALK fusions. Our study suggested that CTC numbers can be used as a preliminary determinant for EGFR mutations to decide if further histological examination is required.

We summarized some studies on CTC numbers and PFS in lung cancer (S4 Fig). These studies used different cut-off values for CTC numbers (from 2 to 362 CTCs) to divide samples into favorable and unfavorable groups [14, 27–32]. The total average PFS of the favorable and unfavorable groups were 8.16 months (from 4.9 to 11.1 months) and 5.43 months (from 4.3 to 6.8 months), respectively, for a total of 520 samples. In this study, we divided the subjects into favorable and unfavorable groups by CTC numbers ≤ 5/7.5 ml. Our study showed a longer PFS for both the favorable and unfavorable groups than did the previous reports. In addition, 4 of 7 reports showed a statistically significant difference in the PFS between favorable and unfavorable groups [14, 27–32]. Our results support the opinion that patients with higher CTC numbers would have worse disease progression than patients with lower CTC numbers.

Compared to traditional CTC detection systems, the approach used in this study has considerable advantages. However, it still has some limitations. For instance, we observed that the CTC count was lower than 5/7.5 ml in a single analysis in two patients, while imaging and physical examination showed disease progression. In addition, we only performed the CTC analysis in patients with NSCLC. Control analyses in healthy subjects or in patients with benign disease were not included, which prevented the determination of the sensitivity and specificity for CTC analysis in NSCLC. Additionally, other biomarkers such as serum CEA and CYFRA 21–1 levels [33], which could also be used for NSCLC detection, were not evaluated simultaneously with CTC detection. Compared with other technologies, the possible advantages or disadvantages of CTC analyses may be better reflected.

In conclusion, the assessment of NSCLC cannot be performed using a single CTC analysis. The clinical value is more significant in the continuous analysis of CTC numbers, as well as the cross-validation of other indexes and imaging results.

## Supporting information

S1 Fig Schematic representation. A. The sample collection scheme with timelines of chemotherapy. B. The analytical workflow of CTC detection.
(PDF)

S2 Fig The detection rate of CTC analyses. The detection rates of CTCs in samples spiked with 50, 100 and 200 tumor cells were higher than 80%.
(PDF)

S3 Fig The summary of CTC numbers in NSCLC patients from published articles. The CTC number is shown as the mean ± SD in results labelled with the “a” tag. The CTC number is shown as the median and range in the results shown without the “a” tag. The results from CTC-positive samples are shown with the “b” tag. The results from both CTC-positive and CTC-negative samples are shown without the “b” tag.
(PDF)

S4 Fig Summary of PFS in the favorable and unfavorable groups of lung cancer from published articles. Blue, favorable group. Dark red, unfavorable group.
(PDF)

S1 Table Primers, probes and controls for detecting EGFR ^E19del/L858R^ mutations. (PDF)
