# Supplementary material for: The application of nano-enrichment in CTC detection and the clinical significance of CTCs in non-small cell lung cancer (NSCLC) treatment
Source: PLoS One. 2019 Jul 25;14(7):e0219129. doi: 10.1371/journal.pone.0219129 (PMC6657845; doi:10.1371/journal.pone.0219129)
Supplement: S1 Fig — A. The sample collection scheme with timelines of chemotherapy. B. The analytical workflow of CTC detection. (PDF) [file pone.0219129.s001.pdf]

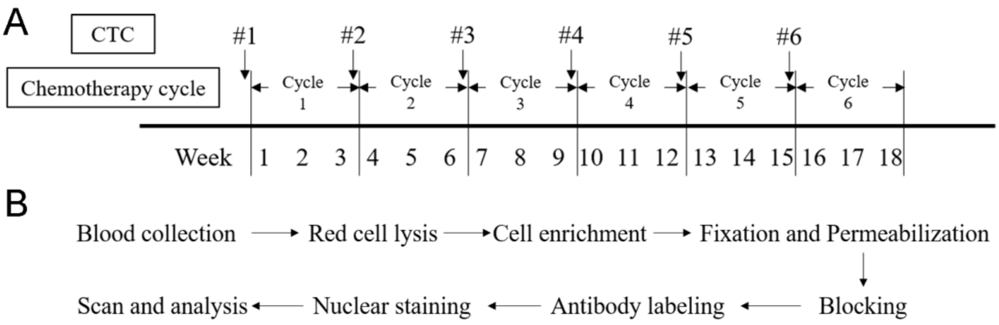

S1 Fig. Schematic representation. A. The sample collection scheme with timelines of chemotherapy. B. The analytical workflow of CTC detection
