# Supplementary material for: The application of nano-enrichment in CTC detection and the clinical significance of CTCs in non-small cell lung cancer (NSCLC) treatment
Source: PLoS One. 2019 Jul 25;14(7):e0219129. doi: 10.1371/journal.pone.0219129 (PMC6657845; doi:10.1371/journal.pone.0219129)
Supplement: S2 Fig — The detection rates of CTCs in samples spiked with 50, 100 and 200 tumor cells were higher than 80%. (PDF) [file pone.0219129.s002.pdf]

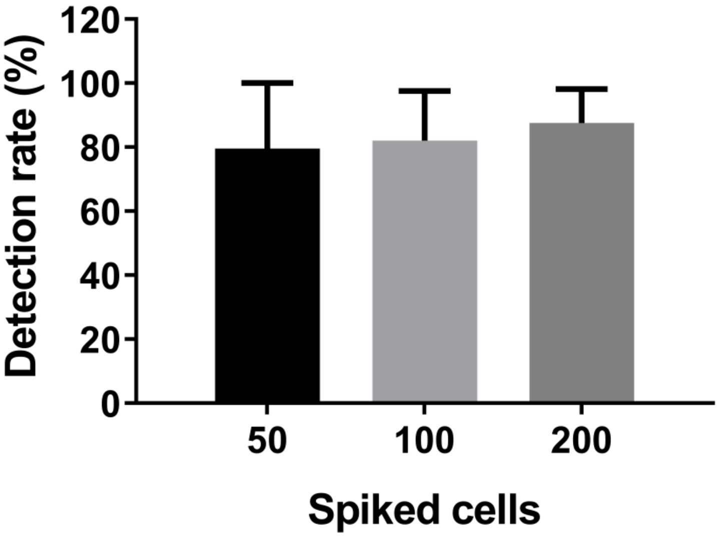

S2 Fig. The detection rate of CTC analyses. The detection rates of CTCs in samples spiked with 50, 100 and 200 tumor cells were higher than 80%.
