# Supplementary material for: The application of nano-enrichment in CTC detection and the clinical significance of CTCs in non-small cell lung cancer (NSCLC) treatment
Source: PLoS One. 2019 Jul 25;14(7):e0219129. doi: 10.1371/journal.pone.0219129 (PMC6657845; doi:10.1371/journal.pone.0219129)
Supplement: S3 Fig — The CTC number is shown as the mean ± SD in results labelled with the “a” tag. The CTC number is shown as the median and range in the results shown without the “a” tag. The results from CTC-positive samples are shown with the “b” tag. The results from both CTC-positive and CTC-negative samples are shown without the “b” tag. (PDF) [file pone.0219129.s003.pdf]

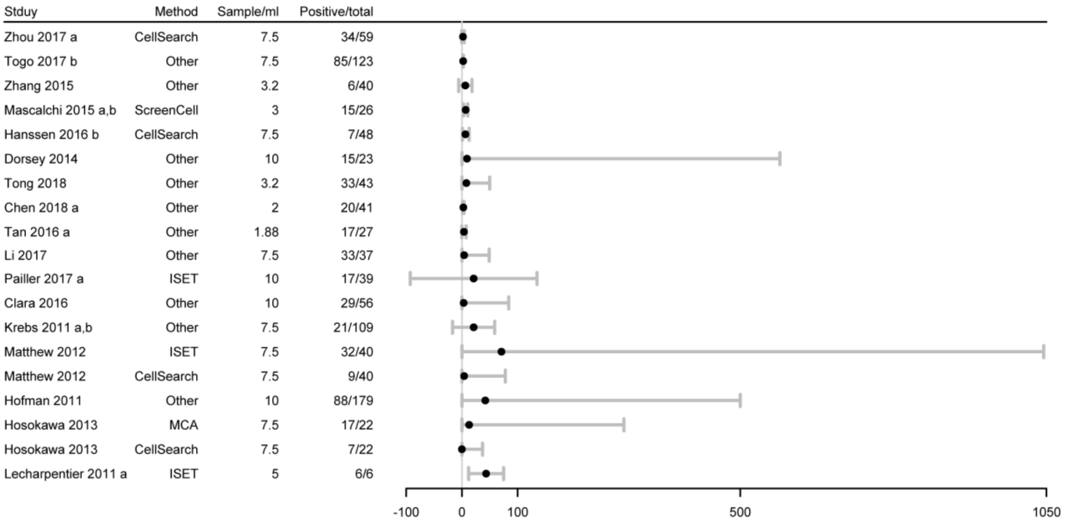

S3 Fig. The summary of CTC numbers in NSCLC patients from published articles. The CTC number is shown as the mean ± SD in results labelled with the “a” tag. The CTC number is shown as the median and range in the results shown without the “a” tag. The results from CTC-positive samples are shown with the “b” tag. The results from both CTC-positive and CTC-negative samples are shown without the “b” tag.
