# Supplementary material for: The application of nano-enrichment in CTC detection and the clinical significance of CTCs in non-small cell lung cancer (NSCLC) treatment
Source: PLoS One. 2019 Jul 25;14(7):e0219129. doi: 10.1371/journal.pone.0219129 (PMC6657845; doi:10.1371/journal.pone.0219129)
Supplement: S4 Fig — Blue, favorable group. Dark red, unfavorable group. (PDF) [file pone.0219129.s004.pdf]

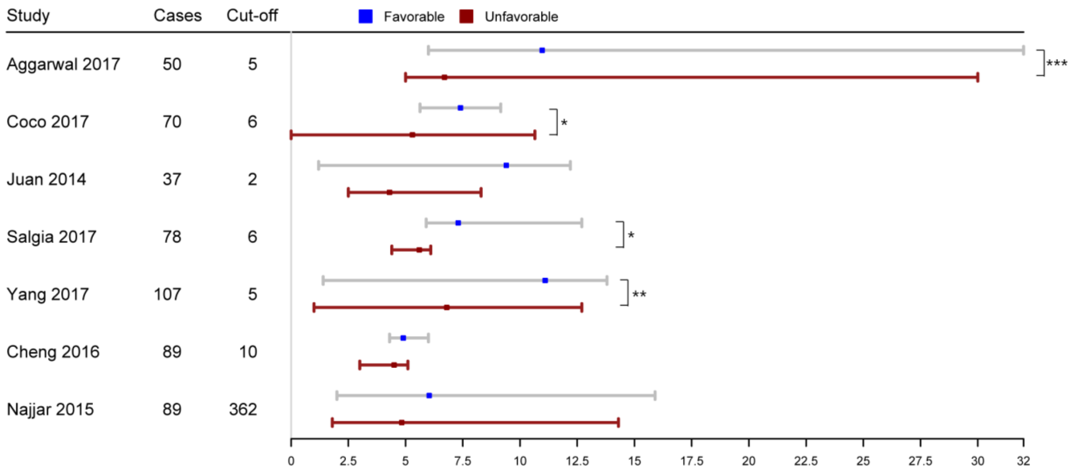

S4 Fig. Summary of PFS in the favorable and unfavorable groups of lung cancer from published articles. Blue, favorable group. Dark red, unfavorable group.
