# Supplementary material for: The application of nano-enrichment in CTC detection and the clinical significance of CTCs in non-small cell lung cancer (NSCLC) treatment
Source: PLoS One. 2019 Jul 25;14(7):e0219129. doi: 10.1371/journal.pone.0219129 (PMC6657845; doi:10.1371/journal.pone.0219129)
Supplement: S1 Table — (PDF) [file pone.0219129.s005.pdf]

S1 Table. Primers, probes and controls for detecting EGFR $^{E19del/L858R}$ mutations.

Exon	Primers and probes (5' to 3')
19(del)	Forward: ACT GGG CAG CAT GTG GCA<br>Reverse 1: CTT GTT GGC TTT CGG TTC<br>Reverse 2: TGT TGG CTT TCG GAA CCT<br>Reverse 3: TGG CTT TCG GAG ATG CCT<br>Reverse 4: GTT GGC TTT CGG AGA TTC<br>Reverse 5: GGC TTT CGG AGA TAT TTT<br>Reverse 6: GGC TTT CGG AGA TGT TTT<br>Reverse 7: TGT TGG CTT TCG GAG ATG CC<br>Reverse 8: TGG CTT TCG GAG ATG G<br>Reverse 9: GCT TTC GGA GAT GTT GG<br>Probe: FAM-AGA GTC CCT ATG ACA GAG AGA GAA GG-BHQ1
21（Leu858R）	Forward: TCA GGG CAT GAA CTA CTT GG<br>Reverse 1: CAC CCA GCT GTT TGG CAC<br>Reverse 2: CAC CCA GCA GTT TGG CACFAM-TGA TCT TGA CAT GCT GCG GTG T-BHQ1
PCR Control	Forward: TGT CCT GGC ACC CAA G<br>Reverse: CAG AGA CAA GGG TCA CCT CA<br>Probe: FAM-TGG AAA GCA GTG CCA GAC ATG G-BHQ1
